# MOSSNet: multiscale and oriented sorghum spike detection and counting in UAV images

**DOI:** 10.3389/fpls.2025.1526142

**Published:** 2025-08-28

**Authors:** Jianqing Zhao, Zhiyin Jiao, Jinping Wang, Zhifang Wang, Yongchao Guo, Ying Zhou, Shiyi Chen, Wenjie Wu, Yannan Shi, Peng Lv

**Affiliations:** ^1^ Key Laboratory for Climate Risk and Urban-Rural Smart Governance, School of Geography, Jiangsu Second Normal University, Nanjing, China; ^2^ Institute of Millet Crops, Hebei Academy of Agriculture and Forestry Sciences, Hebei Branch of National Sorghum Improvement Center, Shijiazhuang, China; ^3^ Key Laboratory of Genetic Improvement and Utilization for Featured Coarse Cereals, Ministry of Agriculture and Rural Affairs, Key Laboratory of Minor Cereal Crops of Hebei Province, Shijiazhuang, China

**Keywords:** sorghum spike, oriented detection boxes, angle feature, deep learning, UAV

## Abstract

**Background:**

Accurate sorghum spike detection is critical for monitoring growth conditions, accurately predicting yield, and ensuring food security. Deep learning models have improved the accuracy of spike detection thanks to advances in artificial intelligence. However, the dense distribution of sorghum spikes, variable sizes and complex background information in UAV images make detection and counting difficult.

**Methods:**

We propose a multiscale and oriented sorghum spike detection and counting model in UAV images (MOSSNet). The model creates a Deformable Convolution Spatial Attention (DCSA) module to improve the network's ability to capture small sorghum spike features. It also integrated Circular Smooth Labels (CSL) to effectively represent morphological features. The model also employs a Wise IoU-based localization loss function to improve network loss.

**Results:**

Results show that MOSSNet accurately counts sorghum spike under field conditions, achieving mAP of 90.3%. MOSSNet shows excellent performance in predicting spike orientation, with RMSEa and MAEa of 14.6 and 12.5 respectively, outperforming other directional detection algorithms. Compared to general object detection algorithms which output horizonal detection boxes, MOSSNet also demonstrates high efficiency in counting sorghum spikes, with RMSE and MAE values of 9.3 and 8.1, respectively.

**Discussion:**

Sorghum spikes have a slender morphology and their orientation angles tend to be highly variable in natural environments. MOSSNet 's ability has been proved to handle complex scenes with dense distribution, strong occlusion, and complicated background information. This highlights its robustness and generalizability, making it an effective tool for sorghum spike detection and counting. In the future, we plan to further explore the detection capabilities of MOSSNet at different stages of sorghum growth. This will involve implementing object model improvements tailored to each stage and developing a real-time workflow for accurate sorghum spike detection and counting.

## 1 Introduction

Sorghum [*Sorghum bicolor* (L.) Moench] is a C_4_ drought tolerant cereal crop widely grown worldwide for food, feed, and biofuels (Bao et al., 2024). It plays a vital role in global food security, especially in arid regions, providing an important source of nutrition for humans and livestock (Liaqat et al., 2024; Hossain et al., 2022). Due to the reduction of arable land and the impact of extreme environments, humans have been committed to maximizing crop yields to feed the growing population (Baye et al., 2022). Sorghum is the fifth cereal crop, and increasing the yield per unit area of sorghum can provide a guarantee for food security. Therefore, accurate estimation of sorghum yield is crucial for farmers and breeders (Khalifa and Eltahir, 2023, Ostmeyer TJ et al., 2022, Wang et al., 2024). Traditional manual sampling methods are not only time consuming and labor intensive, but are susceptible to human error, especially in the case of large-scale planting (Wu et al., 2023). This makes it particularly urgent to achieve efficient and accurate yield estimation (Liang et al., 2024; Wang et al., 2023).

To address these challenges, many studies have used unmanned aerial vehicle (UAV) technology, which has excellent monitoring capabilities, as an effective tool for sorghum yield estimation (Ahmad et al., 2020, Jianqing et al., 2023). Equipped with high-precision sensors, UAVs can quickly capture images of large areas of farmland, greatly improving the speed and accuracy of data collection (Abderahman Rejeb et al., 2022). This aerial perspective provided by UAVs offers comprehensive crop coverage and helps reduce monitoring blind spots. It also enables real-time tracking of crop growth. These capabilities are crucial for identifying potential problems and making timely adjustments (Jin et al., 2021, Zhang et al., 2024a, Zhang et al., 2024b). The flexibility and cost-effectiveness of UAV operations enable efficient deployment in a variety of environments and terrains. These advantages help to significantly reduce labor costs while improving data reliability. As a result, they provide a strong basis for scientific evaluation of sorghum yields (Hasan MM et al., 2018, Perich G et al., 2020).

In recent years, deep learning have achieved groundbreaking success in solving complex problems in a variety of fields (Duan et al., 2024; Yan et al., 2022, Yang et al., 2020; Zhang et al., 2023). The rapid development of deep learning has provided a novel solution for the detection and counting of sorghum spikes (Huang et al., 2023; Sanaeifar et al., 2023). Deep neural networks show significant potential in tasks such as classification, detection and segmentation (Cai et al., 2021, James et al., 2024). While classification methods can effectively categorize images of sorghum spikes, it faces challenges in dealing with complex backgrounds and occlusions (Guo et al., 2018). Segmentation methods achieve precise pixel-level localization but requires significant computational resources, making it less suitable for real-time processing of large datasets (Chenyong et al., 2020; Genze et al., 2022; Malambo et al., 2019, Zarei et al., 2024). Detection methods combine the strengths of both classification and segmentation, efficiently identifying the position and shape of sorghum spikes while demonstrating strong adaptability to varying backgrounds (James et al., 2024; Gonzalo-Martín and García-Pedrero, 2021). Deep learning models improve the accuracy and robustness of sorghum spike detection, especially in complex environments. These approaches effectively meet the real-time processing requirements of large-scale agricultural automation.

Traditional object detection methods based on convolutional neural networks, such as Faster R-CNN, SSD and YOLO, typically use horizontal bounding boxes to localize objects in images. While effective for standard detection tasks, these methods have significant limitations when applied to sorghum panicle detection in UAV imagery. Sorghum panicles have a slender morphology and naturally occur in different orientations due to the influence of wind, gravity and plant structure. Horizontal bounding boxes cannot represent directional information, making it difficult for models to learn accurate spatial features (Gonzalo-Martín and García-Pedrero, 2021; Lin and Guo, 2020; Zhang et al., 2024). Sorghum panicles are small, densely packed and highly overlapping in UAV images. Horizontal boxes often lead to the merging of multiple panicles into a single detection, or the repeated detection of the same panicle in crowded scenes (Duan et al., 2024; Ming et al., 2021). Horizontal boxes tend to contain a large amount of irrelevant background, which interferes with the learning of target-specific features, reducing model accuracy and training efficiency. This problem is exacerbated by the complex field environment, where background elements such as leaves, soil and shadows introduce additional noise (Qiu et al., 2024, Wang et al., 2022; Xue et al., 2024). In recent years, oriented bounding boxes have been used with an additional angular parameter to better represent multi-oriented targets, making some progress in remote sensing and cluttered scene detection. However, simply incorporating orientation into the bounding box is insufficient to address the challenges of small target size, dense overlap, and complex spatial relationships. In UAV imagery, such approaches still struggle with inadequate feature extraction and poor modelling of object interactions, resulting in frequent false positives and missed detections (Sanaeifar et al., 2023). It is therefore critical to develop a detection model that is not only orientation-aware, but also capable of fine-grained feature extraction and robust spatial reasoning.

To address these challenges, this study integrates sorghum spike orientation information into convolutional neural networks and uses oriented bounding boxes to identify their spatial locations. We propose a multiscale and orientation-aware sorghum spike detection model for UAV images. This model is designed to effectively address the detection challenges posed by the complex morphology and significant occlusions commonly found in sorghum spikes.

## 2 Materials and methods

### 2.1 UAV images

**Figure 1:**
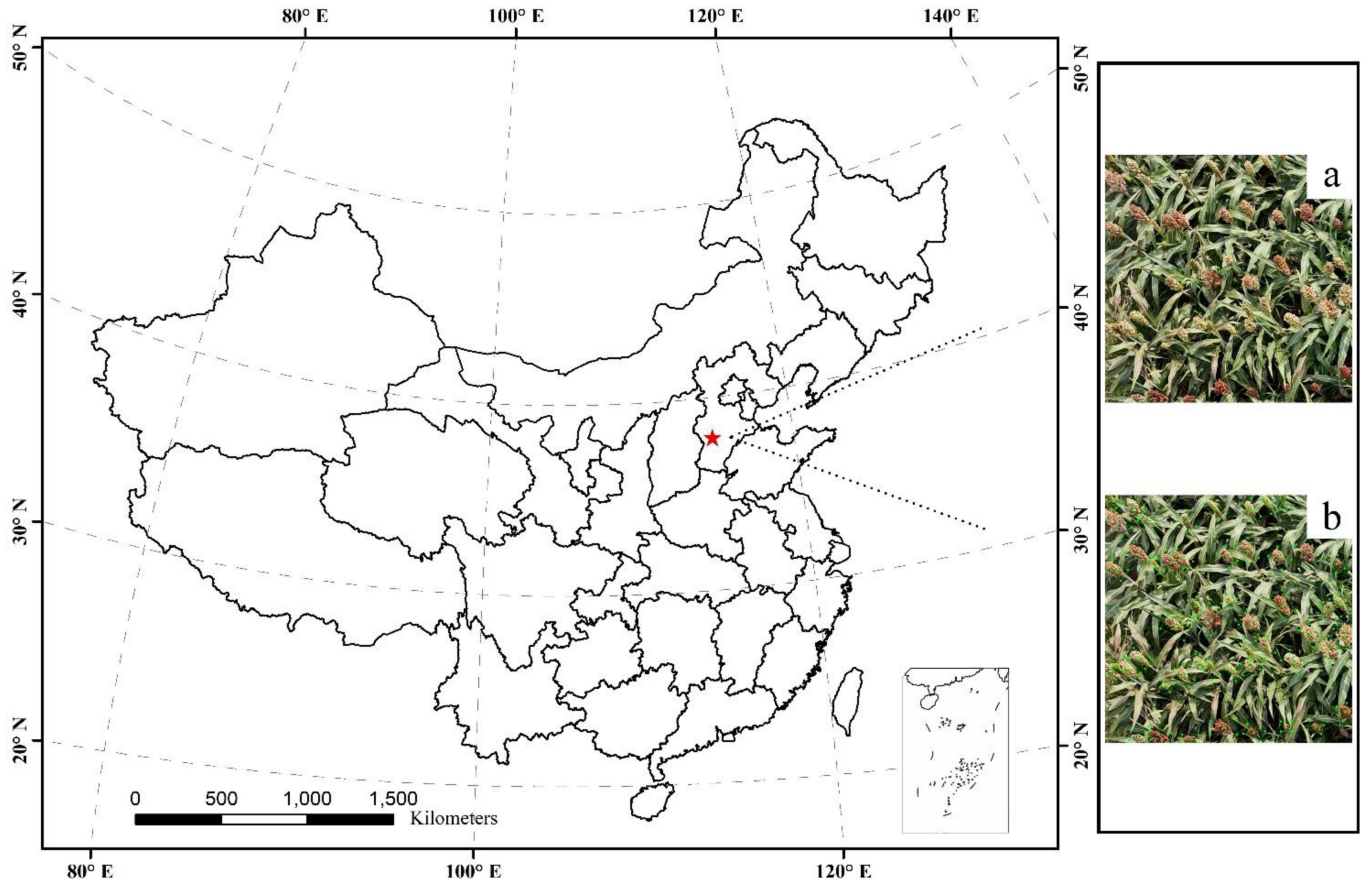
Research area. **(a)** UAV image, **(b)** Labeled image.

The experiment was conducted in 2024 at the Qiema Experimental Station in Shijiazhuang, Hebei Province, China, with sorghum variety JN 4 planted in 3 plots of 900 m^2^ each (**Figure 1**). A DJI Phantom 4 drone equipped with a visible light camera was used to capture images of sorghum spikes under sunny conditions between 10 am and 2 pm. The flight altitude was set at 10 meters, with a speed of 1 meters per second. The original drone images had a resolution of 4032×2268 pixels. To increase the efficiency of the model processing, the original images were cropped into 600×600 pixels sub-images. The sorghum spikes were manually annotated using the Rolabelimg tool. This process generated annotation files containing information on each spike’s center point, dimensions, angle, and category.

### 2.2 Dataset preparation

**Figure 2:**
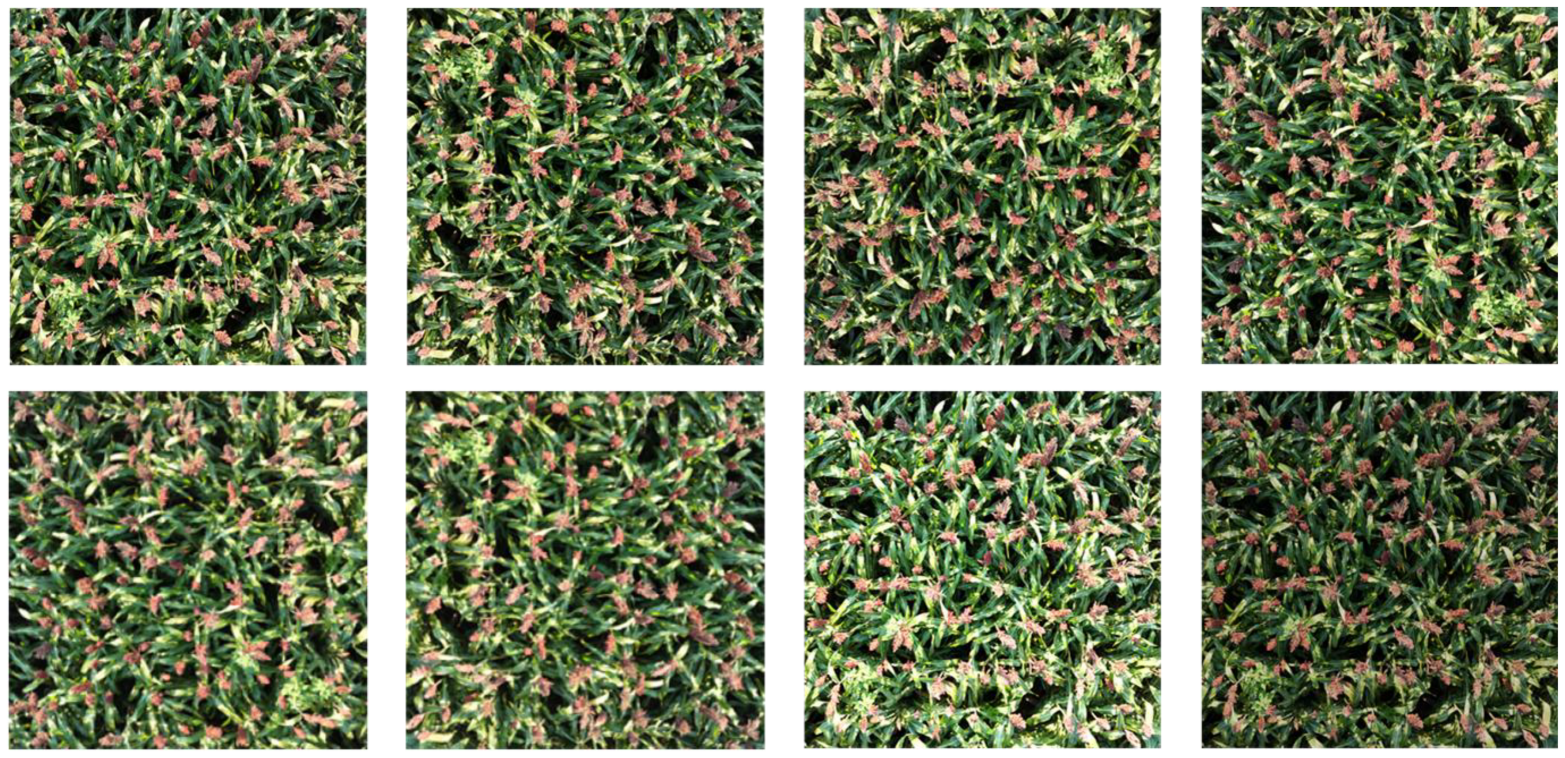
Data augmentation.

Previous studies have shown that rotation and flipping can effectively improve the robustness of deep neural network models. In addition, brightness adjustment helps mitigate the effects of brightness variations caused by changes in ambient lighting and differences in sensors (Alzubaidi L et al., 2021, Zhang et al., 2023). Therefore, we applied data augmentation, including rotation, flipping, and brightness adjustment, to the annotated sorghum spike images and their associated annotation files (**Figure 2**). Specifically, rotation operations included rotating images by 90°, 180° and 270°, while flipping operations included both horizontal and vertical flipping. Brightness adjustment was achieved by increasing and decreasing the image brightness by 10% and 20% respectively. This resulted in a dataset of 6000 images of sorghum spikes. Since the YOLO model inherently extracts approximately 10% of the training set data as an internal validation subset during training. This internal validation procedure continuously can monitor model convergence, early stopping and hyperparameter tuning. The dataset was then randomly divided into training, validation and test sets in a ratio of 6:1:3.

### 2.3 Structure of MOSSNet

**Figure 3:**
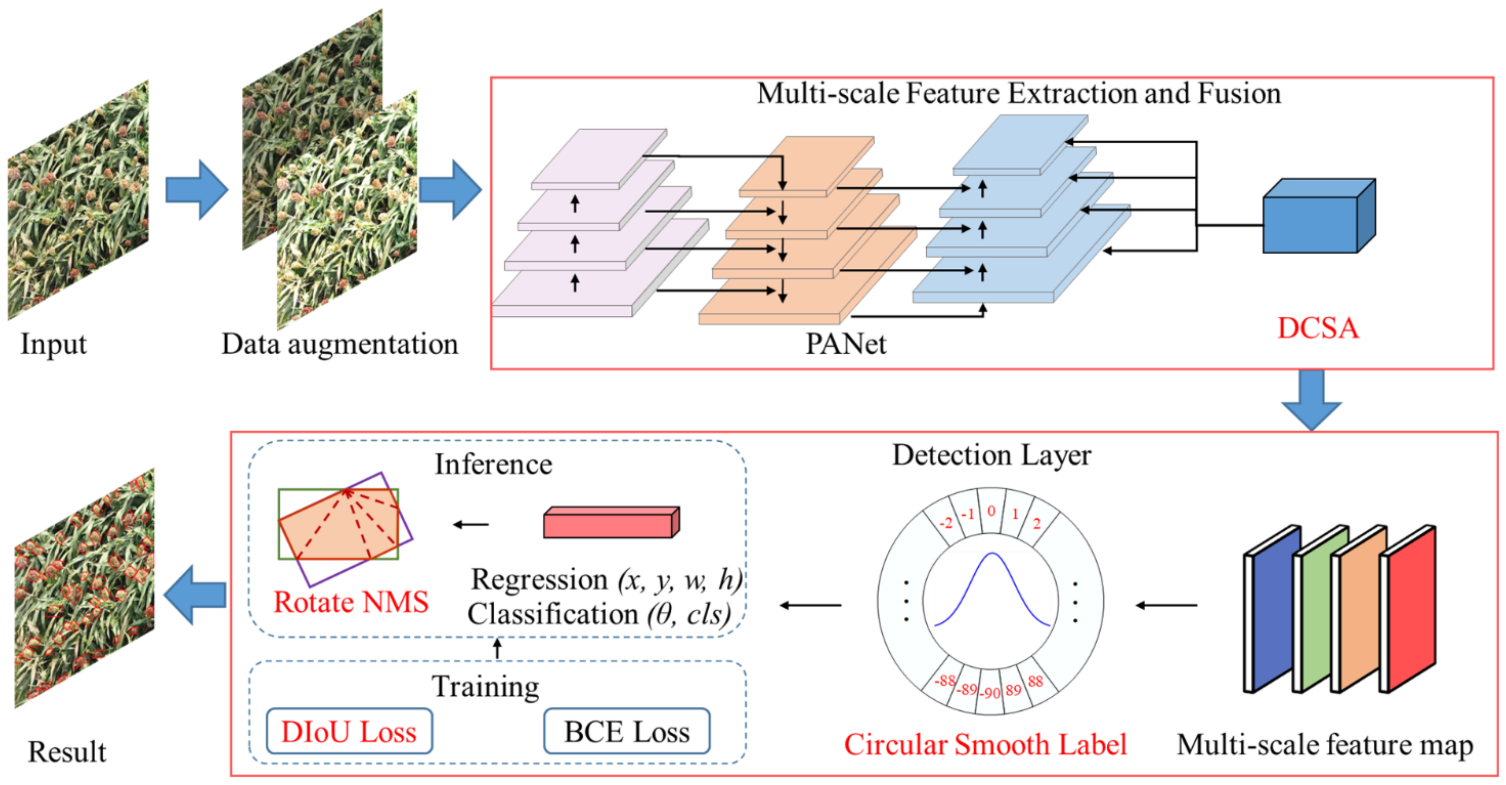
The overall framework of MOSSNet.

MOSSNet is constructed based on YOLOv8. MOSSNet performs feature extraction and fusion on the input image to generate multi-scale feature maps (**Figure 3**). A Deformable Convolution Spatial Attention (DCSA) module is proposed to improve the network’s ability to capture features of small sorghum spikes. An angular feature extraction module is developed by incorporating circular smooth labels. This enables the network to capture the directional information of sorghum spikes, effectively representing their morphological characteristics in UAV images. A Wise-IoU based localization loss is applied to further optimize the network loss for bounding boxes and detection boxes of sorghum spikes in field environments. Finally, duplicate sorghum spike detection boxes are removed by calculating the overlap area of the oriented detection boxes. The size, position, angle, and category information of the remaining detection boxes are then output.

#### 2.3.1 Deformable convolution spatial attention

**Figure 4:**
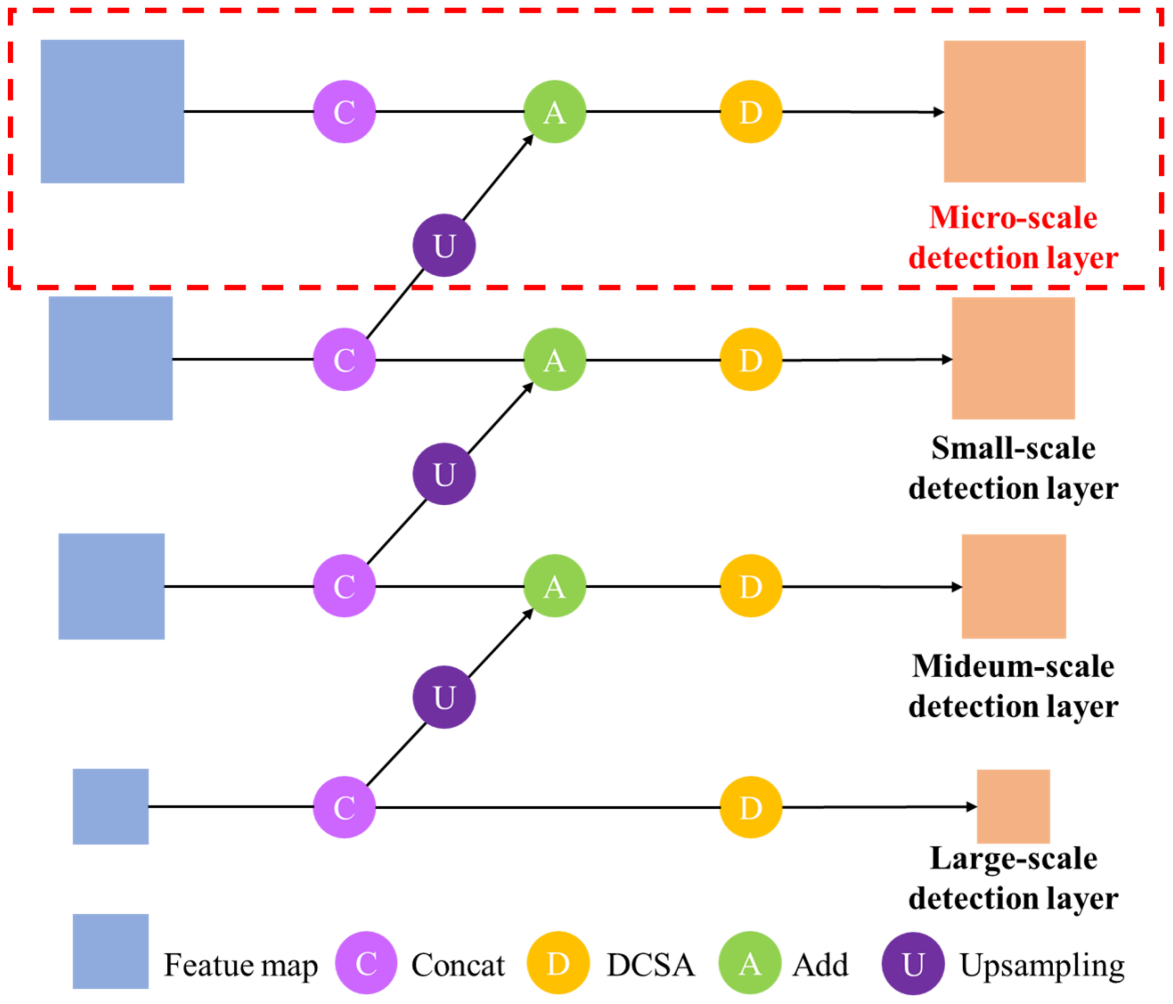
The structure of neck with DCSA.

**Figure 5:**
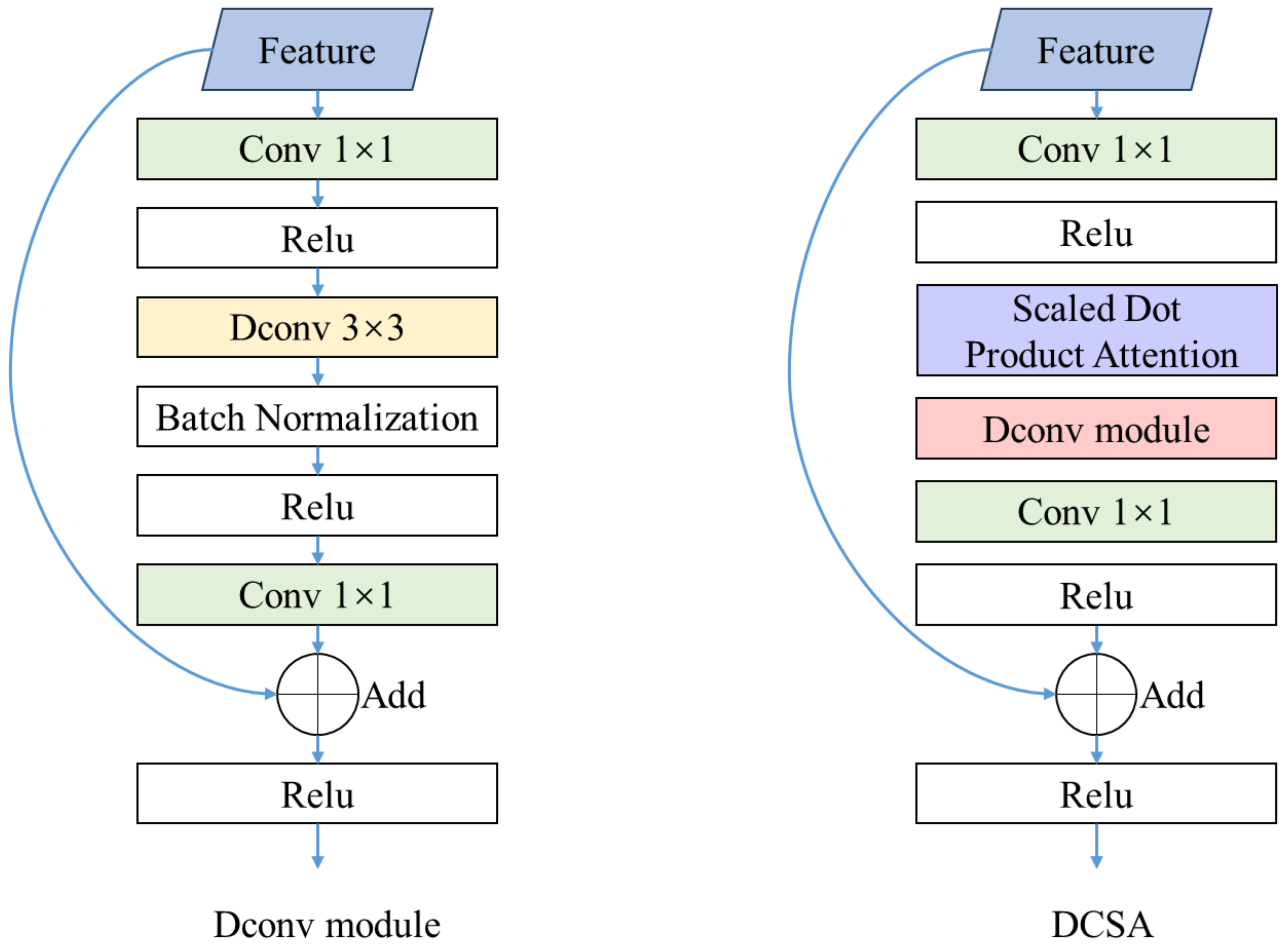
The structure of DCSA and Dconv module.

This study proposes DCSA module to eliminate invalid features generated by traditional convolution operations when processing sorghum spikes of different shapes and sizes (**Figure 4**). The DCSA module has two main components. First, deformable convolution (Dai et al., 2017) is employed to overcome the limitations of conventional convolution in handling rectangular shapes. It allows the network to adaptively extract effective features based on the size of the sorghum spikes. (**Figure 5**). Second, the DCSA integrates deformable convolution with the Scaled Dot-Product Attention mechanism (Lovisotto et al., 2022). Notably, the scaled dot-product attention mechanism enhances the model’s ability to focus on color-dimension features, thereby improving its adaptability to the color variations exhibited by sorghum spikes at different growth stages. By embedding this combination in the multi-scale feature extraction and fusion part of the network, the feature extraction capabilities are significantly enhanced. (**Figure 5**). The DCSA introduces a micro-scale detection layer branch designed to generate feature maps optimized for small sorghum spikes. These feature maps have a resolution of one quarter of the input image (**Figure 4**), ensuring better detection of multi-scale spikes in different stages.

#### 2.3.2 Angle feature extraction

In this study, the oriented detection box of the sorghum spike is decoupled into a horizontal detection box. This box contains the center of mass coordinates (x, y), length and width information (w, h), angle information (θ), and category information (cls). θ is the angle between the long side of the detection box and the x-axis, and each angle is a category, with a total of 180 categories. Circular smooth labels address the issue of angular periodicity (Yang and Yan, 2020). Based on this, the study defines a periodicity labelling code to measure the angular distance between the oriented detection box and the bounding box using the following formula:


$$CSL(x)=\{\begin{array}{l} g(x)\text{, }\theta -r<x<\theta +r\\ \;0\text{, }otherwise \end{array}$$

where *θ* is the angle of the sorghum spike orientation frame, *g(x)* is the Gaussian function, and *r* is the window radius of the Gaussian function.

#### 2.3.3 Optimized loss function

The IoU-based localization loss only considers the scenario where the detection frame and the bounding box intersect, without taking into account the complex spatial relationship between the two frames (Rezatofighi et al., 2019). This study is based on Wise IoU (Tong et al., 2021), which allows the evaluation of the spatial overlap between the bounding boxes. It focuses not only on the area of overlap, but also on the differences in shape and position between the bounding boxes. The formula is as follows:


$$L_{WIoU}=\alpha \cdot L_{IoU}$$

$$L_{IoU}=\sum_{i=0}^{S^{2}}\sum_{j=0}^{B}I_{ij}(1-IoU)$$

$$\alpha =\exp(\frac{{\left(GT_{x}-B_{x}\right)}^{2}+{\left(GT_{y}-B_{y}\right)}^{2}}{{\left(GT_{w}+GT_{h}\right)}^{2}})$$

where *S* ^2^ is the feature map size output from the detection layer and *B* is the detection frame. $GT_{x}$ , $GT_{y}$ , $GT_{h}$ and $GT_{w}$ are the center coordinates, length and width of the sorghum spike bounding box respectively. $B_{x}$ and $B_{y}$ are the center coordinates of the sorghum spike detection frame.

#### 2.3.4 Duplicate detection boxes deleted

**Figure 6:**
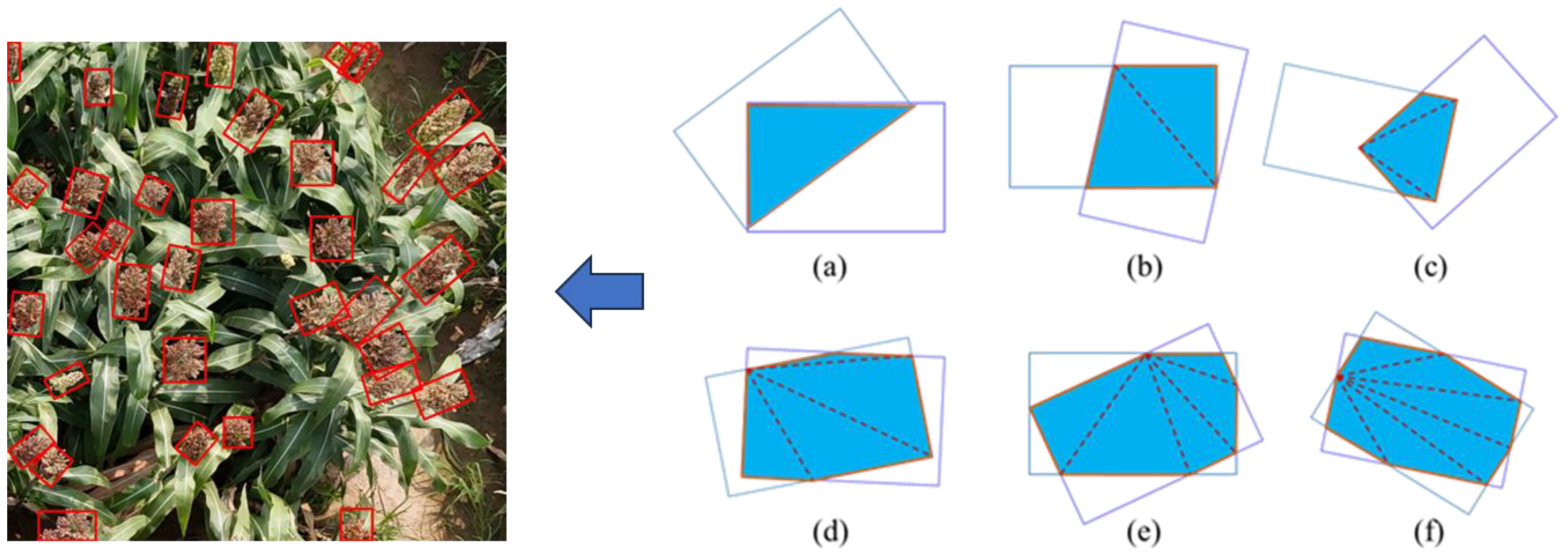
Area calculation for two overlapping oriented detection boxes. **(a)** intersecting graph is a triangle, **(b)** intersecting graph is a quadrilateral, **(c)** intersecting graph is a pentagon, **(d)** intersecting graph is a hexagon, **(e)** intersecting graph is a heptagon, **(f)** intersecting graph is an octagon.

The traditional Non-Maximum Suppression (NMS) method is primarily used to compute the Intersection over Union (IoU) between horizontal bounding boxes. However, this approach presents challenges when applied to the oriented bounding boxes of sorghum spikes (Neubeck and Gool, 2006). To address this issue, this study divides the overlapping region between the sorghum detection box and the bounding box into multiple triangles that share the same vertices (**Figure 6**). The total overlapping area is obtained by calculating and summing the area of each triangle. In addition, the ratio of the overlapping area to the sum of the areas of the two bounding boxes is defined. The bounding box with the highest ratio is retained, while the others are discarded, resulting in the final detection result. **Figure 6** illustrates the geometric principle for calculating the overlapping area between two oriented detection boxes.

## 3 Experiment setup and evaluating indicators

### 3.1 Experiment settings

The deep network models were trained and tested on a server equipped with an Intel^®^ Xeon Platinum 8268 CPU, NVIDIA TITAN V graphics processor (12 GB of graphics memory), and 500 GB of RAM, running Ubuntu 16. Considering the dataset size, model parameters, and computational resources, model training was performed with a learning rate of 0.001, a batch size of 32, a weight decay value of 1e-4, and a momentum of 0.9.

### 3.2 Evaluating indicators

This study evaluates the accuracy of MOSSNet and other deep learning network models in recognizing and counting sorghum spikes using five indicators grouped into three categories. Specifically, a mean Average Precision (mAP) is used to evaluate the accuracy of MOSSNet and specialized detection models in detecting the number and location of sorghum spikes. *RMSE_a_* and *MAE_a_* are used to evaluate the accuracy of the models in estimating the angle of sorghum spike. Meanwhile, *RMSE* and *MAE* are used to assess the performance of MOSSNet compared to general object detection methods for spike detection.

The mAP represents the mean accuracy across all categories within a recall range of 0 to 1, with higher values indicating better detection accuracy. In this study, the only object category is sorghum spikes and the formula for calculating mAP is as follows:


$$\text{mAP}=\int_{0}^{1}P(R)dR$$

where *P* and *R* are the precision and recall, respectively, defined as follows:


$$P=\frac{TP}{FP+TP}$$

$$R=\frac{TP}{FN+TP}$$

*TP* and *FP* are the number of correctly and incorrectly detected sorghum spikes, and *FN* is the number of undetected sorghum spikes.

*RMSE_a_* and *MAE_a_* are used to evaluate the difference between the angle of the sorghum spike detection frame and the angle of the bounding box.


$$RMSE_{a}=\sqrt{\frac{1}{F}\sum_{i=1}^{F}{\left(p_{i}^{n}-q_{i}^{n}\right)}^{2}}$$

$$MAE_{a}=\frac{1}{F}\sum_{i=1}^{F}\left|p_{i}^{n}-q_{i}^{n}\right|$$

where *F* is the number of images for which the calculation was performed. $p_{i}^{n}$ is the predicted angle of the nth sorghum spike in image *i* of the model calculation. $q_{i}^{n}$ is the true angle of the corresponding sorghum spike.

As the output of the generalized one-stage and two-stage object detection methods is a horizontal detection box, it is not possible to use the mAP for comparison with MOSSNet. For the collected UAV images, we use manual annotation, where each sorghum spike was surrounded by a bounding box, and each predicted box from the model was correspondingly treated as a spike. To assess the accuracy of the model in predicting the total number of spikes and the associated variance, *RMSE* and *MAE* are used to compare the difference between the total number of spikes in the annotations and that predicted by the model.


$$RMSE=\sqrt{\frac{1}{F}\sum_{i=1}^{F}{\left(a_{i}-t_{i}\right)}^{2}}$$

$$MAE=\frac{1}{F}\sum_{i=1}^{F}\left|a_{i}-t_{i}\right|$$

where *t* is the number of sorghum spikes included in the model-calculated image *i*, and *a* is the number of manually labeled sorghum spikes in image *i*.

## 4 Results

This study evaluates the performance of MOSSNet in sorghum spike detection against R2CNN (Jiang et al., 2017), R3DET (Yang et al., 2019), RRPN (Liu JH et al., 2017), RIDET (Ming et al., 2021), SCRDET (Yang et al., 2019) and RSDET (Qian et al., 2021) using metrics such as mAP, *RMSE_a_* and *MAE_a_*. These oriented object detection methods are primarily designed to detect rotated objects in remote sensing imagery, and their core mechanisms can be grouped into two categories. The first involves the incorporation of rotated bounding box regression (e.g., R2CNN, RRPN, R3Det), either at the proposal generation stage or at the regression stage, to model the angular information of objects. The second focuses on feature refinement and structure optimization (e.g. SCRDet, RIDet, RSDet) to improve detection performance for small objects or those with large angular variations in complex backgrounds. The results show that MOSSNet achieves a mean average precision (mAP) of 90.3% (**Table 1**), outperforming the other methods in terms of detection accuracy. MOSSNet also achieved the highest precision in predicting sorghum spike orientation, with *RMSE_a_* and *MAE_a_* of 14.6 and 12.5 respectively. MOSSNet’s frame per second (FPS) reached 35, demonstrating its ability to efficiently detect the orientation of sorghum spikes in UAV imagery.

**Table 1:** Comparison between the proposed method and other oriented object detection approaches.

Method	mAP (%)	RMSE_a_	MAE_a_	FPS
Proposed	90.3	14.6	12.5	35
R2CNN	67.1	46.9	39.5	24
R3DET	76.5	31.3	25.3	32
RRPN	71.4	42.3	36.8	28
RIDET	84.7	19.5	18.3	20
SCRDET	78.3	26.6	21.2	27
RSDET	75.2	32.3	28.4	25

RMSE_a_ and MAE_a_ are used to evaluate the difference between the angle of the sorghum spike detection frame and the angle of the bounding box. The subscript lowercase letter ‘a’ means that they are different from RMSE and MAE.

**Figure 7:**
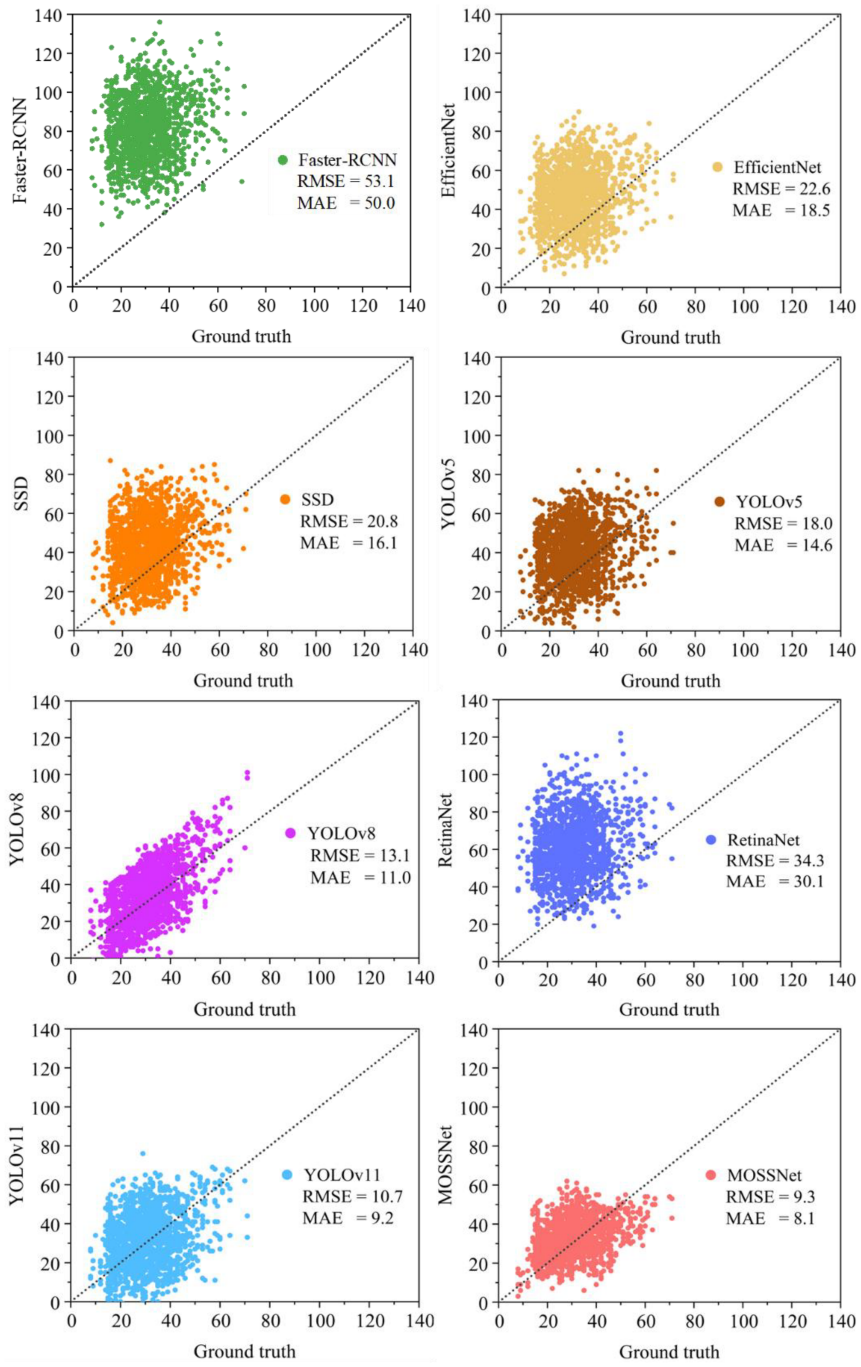
Comparison of the number of spikes labeled on the images by MOSSNet and other methods.

Furthermore, this study compared MOSSNet with several mainstream horizontal bounding box object detection methods, including Faster RCNN (Ren et al., 2017), EfficientNet (Tan and Le, 2019), RetinaNet (Lin et al., 2017), SSD (Wei et al., 2016), YOLOv5, YOLOv8, and YOLOv11 (**Figure 7**). In **Figure 7**, the x-axis represents the actual number of spikes in a given image, while the y-axis represents the number of spikes predicted by the model. Each point on the scatterplot thus corresponds to an image that pairs its labelled spike count with the model’s predicted spike count. The distribution of these points around the diagonal visually illustrates the overall accuracy and error distribution of the model. The results show that each image contains on average about 30 sorghum spikes and MOSSNet still achieves the highest accuracy among all methods, with an *RMSE* of 9.3 and an *MAE* of 8.1. Comparing this benchmark with the outputs of different detection models, Faster R-CNN, EfficientNet, RetinaNet, SSD, YOLOv5, YOLOv8, YOLOv11, and the proposed MOSSNet detected approximately 81, 60, 43, 40, 34, 46, 33, and 31 spikes per image, respectively. These results indicate that some models significantly overestimate the number of spikes, whereas MOSSNet produces spike counts closest to the ground truth, demonstrating higher detection accuracy and better model alignment. As an improved model based on YOLOv8, MOSSNet reduced the *RMSE* by 29% and the *MAE* by 26% compared to YOLOv8. This study also tested the latest YOLOv11, released this year, and the results showed that MOSSNet reduced the *RMSE* by 13% and the *MAE* by 12% compared to YOLOv11. These results show that the proposed MOSSNet performs well in detecting and counting sorghum spikes in complex field environments.

## 5 Discussion

### 5.1 The influence of angle and size distribution for the detection

**Figure 8:**
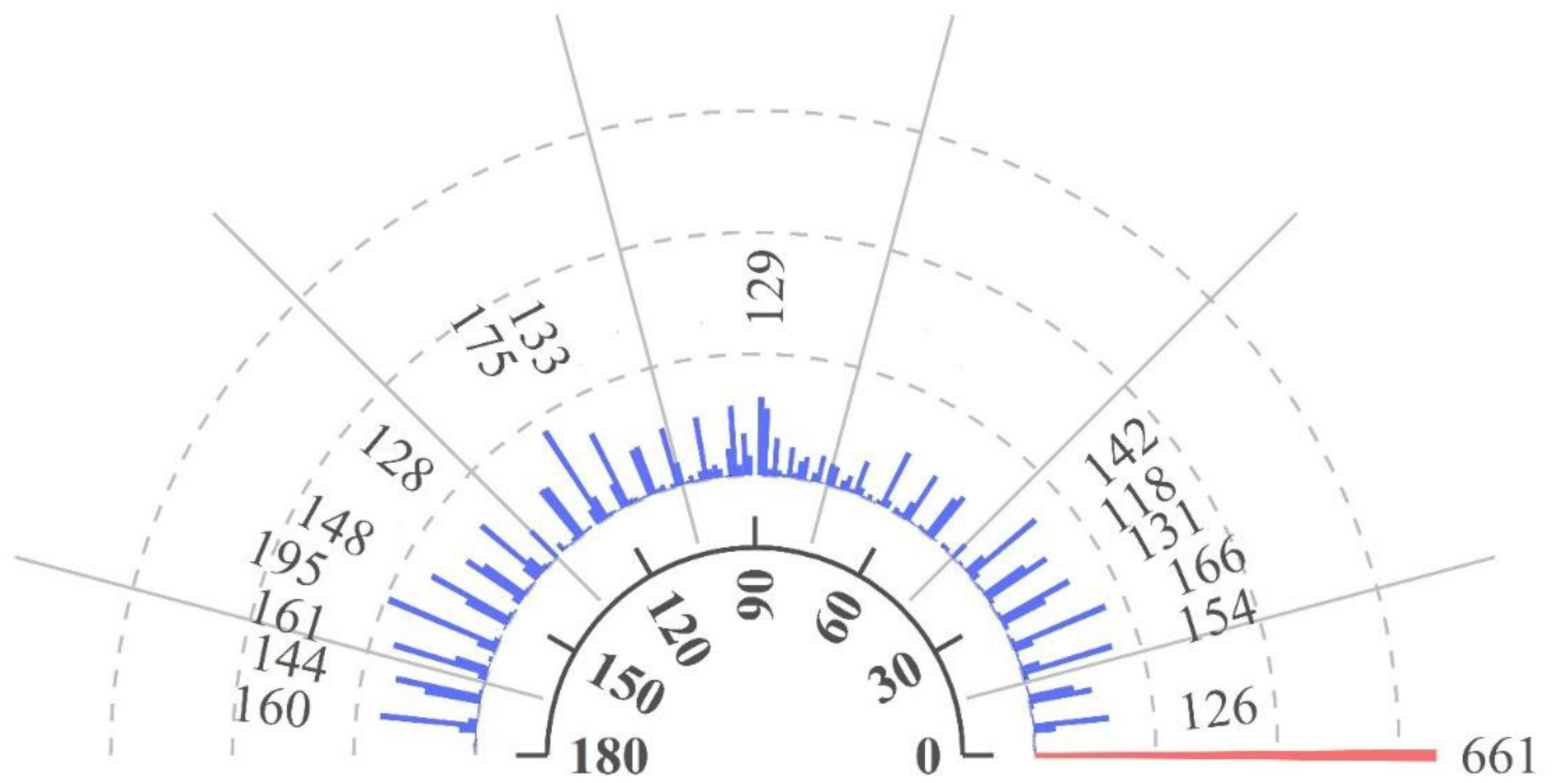
Classification for angles of sorghum spikes.

The number, length and width of crop spikes are critical parameters for crop growth monitoring and yield prediction, which rely heavily on spike angle information in images (Chang et al., 2021; Li et al., 2022). Traditional non-deep learning methods using features such as size, texture, color and morphology combined with operators such as Harris have successfully measured spike morphology. These methods highlight the importance of angular features for effective spike detection (AB et al., 2015, Guo et al., 2016). By manually annotating sorghum spike angles, MOSSNet introduces and extracts spike angle features, providing a more accurate description of sorghum spike morphology in UAV imagery. This study analyses UAV image data to quantify the number of sorghum spikes at different angles. **Figure 8** shows the analysis of UAV images used to quantify the number of sorghum spikes at various angles. Specifically, the horizontal axis, which ranges from 0 to 180, represents the angles at which the sorghum spikes are rotated in the images. This allows their morphological features to be captured. The red and blue lines in the figure show the number of sorghum spikes detected at specific angles. At an angle of 0°, the corresponding number of detected sorghum spikes is 661. The numbers labelled in the figure represent the exact number of spikes at intervals based on a threshold of 100, which emphasizes the differences in spike quantities. These results show that sorghum spikes are unevenly distributed across different angles, appearing mainly between 0°-45°, 90° and 120°- 180°, with the highest number at 0°. This distribution may be influenced by visual preferences and operational habits during manual annotation (Zhao et al., 2022). Consequently, the uneven distribution of spike angles suggests variation in morphology and orientation in UAV imagery based on **Figure 8**. By incorporating directional features, MOSSNet can more accurately capture the true morphology of sorghum spike in UAV images. This improves the model’s ability to provide reliable data for future UAV-based estimation of sorghum spike morphological parameters.

**Figure 9:**
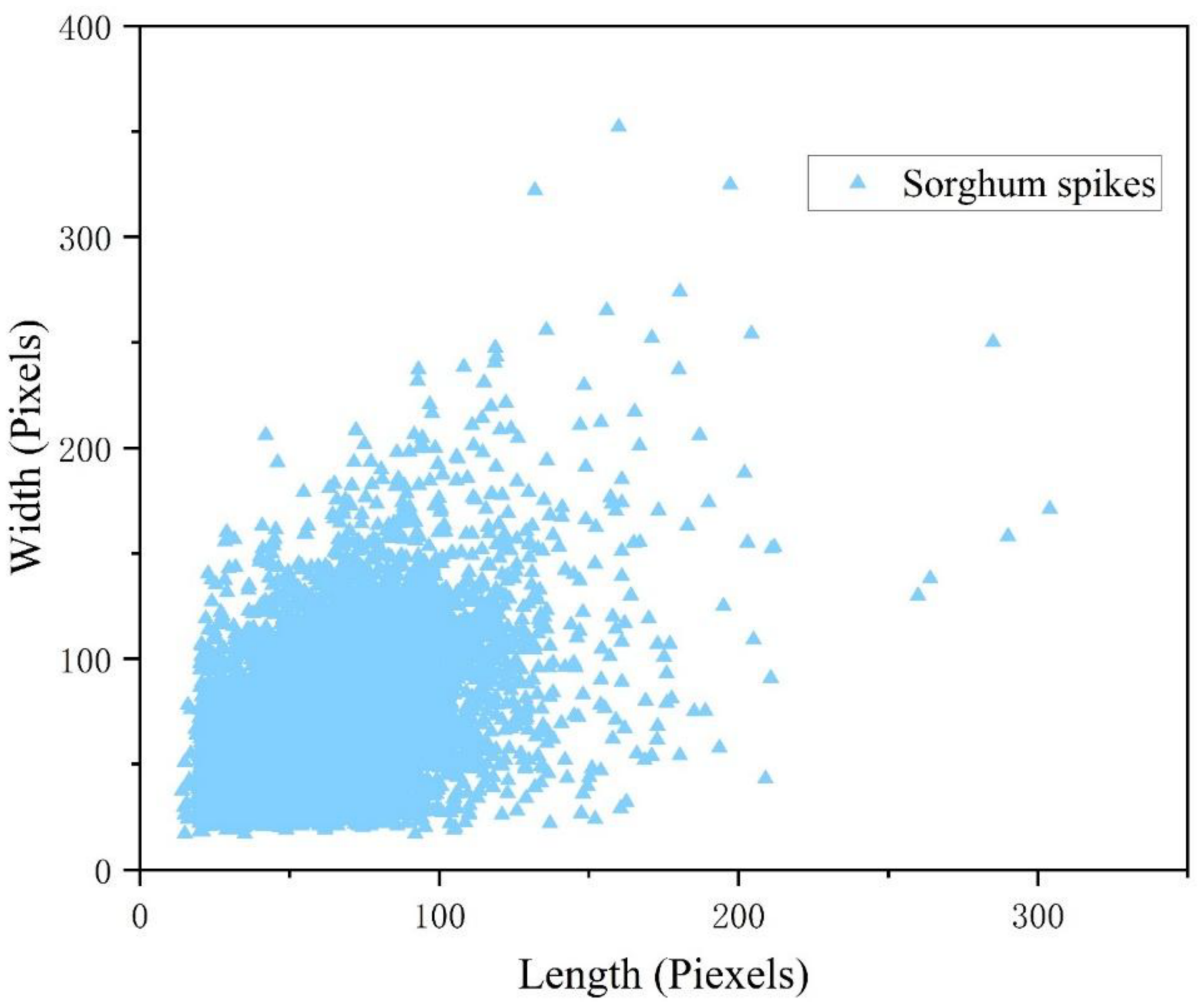
The size distribution of sorghum spikes in UAV images.

In this study, a detailed analysis of the bounding box length, width and area of sorghum spikes in the measured data was conducted (**Figure 9**). The bounding box length and width of sorghum spikes in drone images are generally less than 100 pixels. It indicates that the scale of sorghum spikes is relatively small and falls within the range of small object detection. On the other hand, the study used visible light images with a resolution of 4032×2268 taken by a drone flying at an altitude of 10 meters, resulting in a ground resolution of 0.4 cm/pixel. The combination of observation scale and representation scale determines that the acquired drone images have the characteristics of small size and high density of sorghum spikes. Under these conditions, the pixel area of small objects in the image is relatively small, resulting in blurred image representation, limited extractable features, and weaker feature representation ability (Zhao et al., 2021). Therefore, it is necessary to make improvements to the detection model based on the characteristics of small-sized objects to improve its performance in detecting and representing sorghum spikes.

### 5.2 Ablation study and generalization test

**Figure 10:**
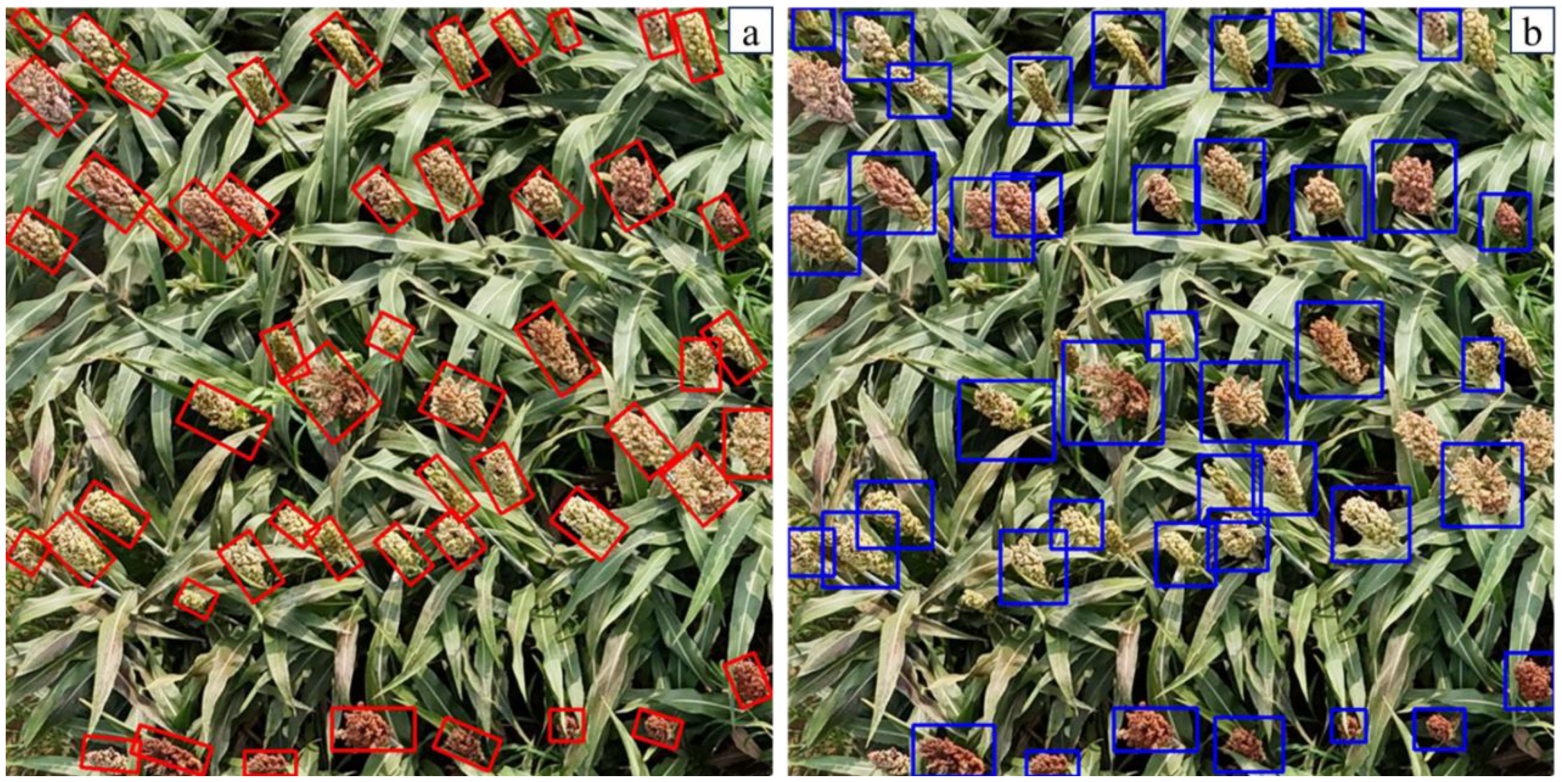
Detection results with MOSSNet **(a)** and the YOLOv8 **(b)**.

**Figure 11:**
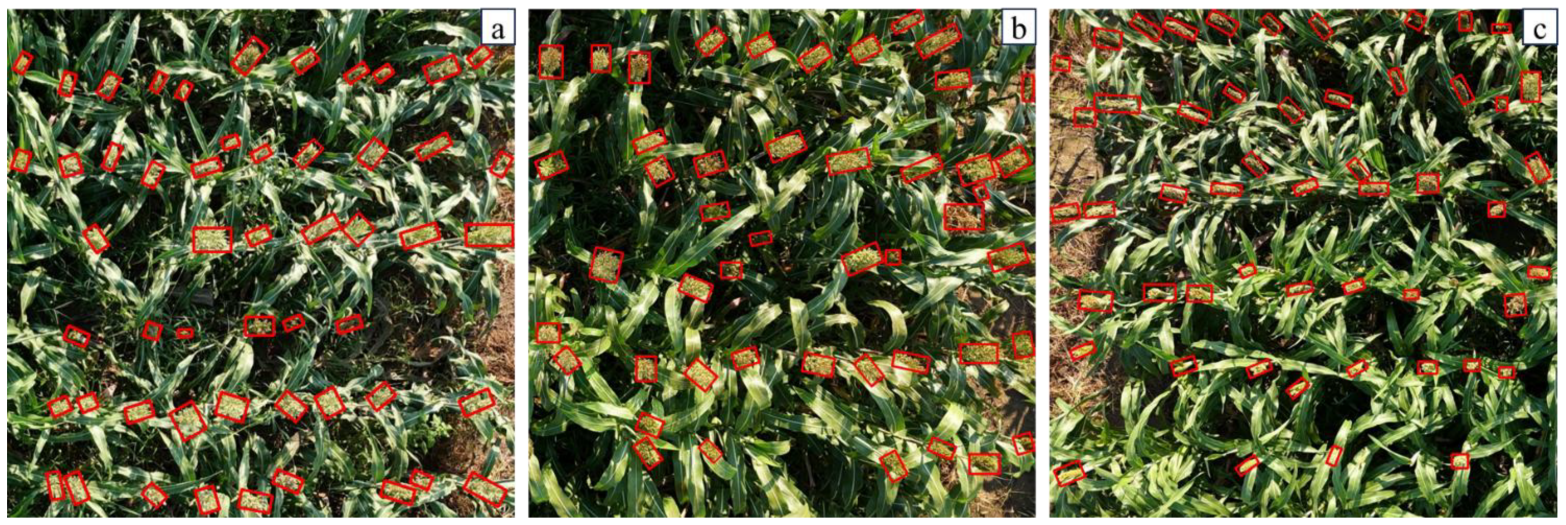
Detection results on different varieties with MOSSNet. **(a)** JN3; **(b)** JN6; **(c)** JN8.

To further investigate the impact of DCSA, CSL and Wise IoU on the experimental results, this study evaluates the effectiveness of these improvements with mAP (**Table 2**). The results show that CSL is the most effective improvement, increasing mAP by 5.5%, while DCSA and Wise IoU contribute increases of 3.9% and 1.6% respectively. The baseline model only transforms sorghum spike angle information into standard categorical features, which is insufficient to capture the relative distances between different angles and the periodic nature of angle features. Comparing the results of oriented and horizontal bounding boxes for sorghum spike detection, it is clear that the inclusion of CSL for oriented detection offers significant advantages. The oriented bounding boxes are better able to capture the morphological features of the sorghum. In addition, they contain less background noise, which improves the overall detection accuracy (**Figure 10**). Without CSL, the horizontal bounding boxes tend to generate significant overlap, which interferes with sorghum spike detection and leads to missed detections. In addition, horizontal bounding boxes containing excessive background not only increase false detections, but also prevent further in-depth analysis of the detection boxes. Using our existing data resources, we conducted additional tests on JN3, JN6 and JN8 and their inbred lines (**Figure 11**). The results show that the proposed model has high generalization and robustness in different backgrounds and field conditions.

**Table 2:** Ablation study of components of MOSSNet.

CSL	DCSA		Wise IoU	mAP (%)
	Dconv	Scaled dot-product attention		
				79.3
√				84.8
√	√			85.9
√	√	√		88.7
√	√	√	√	90.3

Checkmark (√) indicates that the corresponding module is used. The absence of a checkmark indicates that the module is not used.

### 5.3 Optimized pathways for sustainable monitoring of sorghum spikes

Sorghum spikes have a slender morphology and their orientation angles tend to be highly variable in natural environments. Traditional horizontal detection boxes contain a significant number of non-target background pixels, reducing detection accuracy (Sanaeifar et al., 2023). The output does not capture the directional characteristics of rice spikes. The inclusion of complex background information in the horizontal bounding boxes can further degrade the model’s training performance and make it difficult to visualize the detection results (Zhang et al., 2025). Sorghum plants have flexible structures, with dense, interlaced inflorescences that often overlap heavily, making false positives and false negatives more common than in standard object detection tasks. Under the influence of natural wind or UAV rotor wash, sorghum spikes can sway and intertwine, making it difficult to identify individual spikes. Our results show that sorghum spikes are unevenly distributed at different angles, occurring mainly between 0°-45°, 90° and 120°-180°, with the highest concentration at 0°. The model shows excellent adaptability, even in windy conditions. This angular distribution highlights a potential subjective bias in manual counting and serves as a reminder to farmers to consider the angular variations caused by wind, as failure to do so may compromise the accuracy of yield estimation. The model proposed in this study outputs oriented detection boxes with directional features that closely align with each sorghum ear, reducing background interference and improving counting accuracy.

On the other hand, the color of sorghum spikes in UAV images is influenced by the growth stage, which plays a dominant role in the performance of sorghum spike detection methods. This observation suggests that color-based detection approaches are generally used for specific growth stages (Chen H et al., 2024, Ghosal et al., 2019). In this study, the dataset covers both the heading and flowering stages of sorghum and includes several varieties (JN3, JN4, JN6 and JN8). Differences in cultivars and growth stages result in differences in spike color, such as green or brown. Some studies suggest that very early green spikes can exhibit significant differences in shape and size, requiring a larger amount of training data to maintain model generalizability and computational efficiency (Zhang et al., 2022). This study introduced the DCSA module, integrating Deformable Convolution and the Scaled Dot-Product Attention mechanism into the multi-scale feature extraction and fusion processes. The DCSA module combines deformable convolution with a scaled dot-product attention mechanism. The deformable convolution component introduces learnable 2D offsets to the regular sampling grid, enabling the sampling pattern to be deformed freely. This allows the model to focus adaptively on the key spatial features of the panicle regions in the image. Meanwhile, the scaled dot-product attention mechanism enables the model to focus on the most relevant parts of the input, thereby enhancing its ability to model information along the color dimension. Combining adaptive spatial sampling with color feature attention markedly improves the model’s stability in recognizing sorghum panicles across images from different growth stages, effectively mitigating the drop in detection accuracy caused by variation in panicle color. we will also incorporate more rigorous statistical significance analyses in future studies to strengthen the reproducibility and persuasiveness of our results.

In addition, UAV flight parameters directly affect image quality, which has a significant impact on the accuracy of sorghum spike detection and yield estimation. Future research will focus on expanding and refining the sorghum spike dataset. It will also aim to identify the optimal UAV parameters and flight conditions. In addition, images from other growth stages will be incorporated to further improve the accuracy and applicability of the oriented sorghum spike detection model.

The proposed MOSSNet demonstrates significant effectiveness in detecting sorghum spikes of different sizes from UAV images, making it highly valuable for practical applications in field environments. Traditional detection methods struggle to capture the size and morphological characteristics of sorghum spikes in complex environments, especially given the dynamic changes in spike size and shape during the growth process (Guo et al., 2018; Salas Fernandez MG et al., 2017). The DCSA module in MOSSNet accurately captures the characteristics of small and densely spaced spikes. Horizontal detection boxes often contain excessive background information and are susceptible to interference from densely packed spikes (Chen H et al., 2024). By incorporating the CSL module, MOSSNet effectively discriminates the morphological and directional characteristics of sorghum spikes, thereby improving detection accuracy and reliability. In addition, MOSSNet, built with Pytorch, is a deep learning model that can be deployed on cloud servers or edge computing devices. It supports automated, real-time monitoring of sorghum. As a result, it provides farmers with efficient, accurate and informed planting and management strategies.

## 6 Conclusions

This paper proposes a multiscale and oriented sorghum spike detection method in UAV images. Experimental results show that MOSSNet accurately identifies and counts sorghum spike under field conditions, achieving mAP of 90.3%. MOSSNet shows excellent performance in predicting spike orientation, with *RMSE_a_* and *MAE_a_* of 14.6 and 12.5 respectively, outperforming other directional detection algorithms. Com-pared to general object detection algorithms which output horizonal detection boxes, MOSSNet also demonstrates high efficiency in counting sorghum spikes, with *RMSE* and *MAE* values of 9.3 and 8.1, respectively. These results demonstrate the model’s ability to handle complex scenes with dense distribution, strong occlusion, and complicated background information. This highlights its robustness and generalizability, making it an effective tool for sorghum spike detection and counting. In the future, we plan to further explore the detection capabilities of MOSSNet at different stages of sorghum growth. This will involve implementing object model improvements tailored to each stage and developing a real-time workflow for accurate sorghum spike detection and counting.

## Data Availability

The raw data supporting the conclusions of this article will be made available by the authors, without undue reservation.
